# Community-level influences on women's experience of intimate partner violence and terminated pregnancy in Nigeria: a multilevel analysis

**DOI:** 10.1186/1471-2393-12-128

**Published:** 2012-11-14

**Authors:** Diddy Antai, Sunday Adaji

**Affiliations:** 1 Division of Global Health & Inequalities, The Angels Trust - Nigeria, Abuja, Nigeria; 2 Karolinska Institutet, Department of Public Health, Division of Social Medicine, Stockholm, Sweden; 3 Department of Obstetrics & Gynaecology, Ahmadu Bello University Teaching Hospital, PMB 1026, Zaria, Nigeria

**Keywords:** Terminated pregnancy, Community, Multilevel logistic regression analysis, Intimate partner violence, Cross-sectional study, Nigeria

## Abstract

**Background:**

Intimate partner violence (IPV) is a major public health problem with serious consequences for women’s physical, mental, sexual and reproductive health. Reproductive health outcomes such as unwanted and terminated pregnancies, fetal loss or child loss during infancy, non-use of family planning methods, and high fertility are increasingly recognized. However, little is known about the role of community influences on women's experience of IPV and its effect on terminated pregnancy, given the increased awareness of IPV being a product of social context. This study sought to examine the role of community-level norms and characteristics in the association between IPV and terminated pregnancy in Nigeria.

**Methods:**

Multilevel logistic regression analyses were performed on nationally-representative cross-sectional data including 19,226 women aged 15–49 years in Nigeria. Data were collected by a stratified two-stage sampling technique, with 888 primary sampling units (PSUs) selected in the first sampling stage, and 7,864 households selected through probability sampling in the second sampling stage.

**Results:**

Women who had experienced physical IPV, sexual IPV, and any IPV were more likely to have terminated a pregnancy compared to women who had not experienced these IPV types.

IPV types were significantly associated with factors reflecting relationship control, relationship inequalities, and socio-demographic characteristics. Characteristics of the women aggregated at the community level (mean education, justifying wife beating, mean age at first marriage, and contraceptive use) were significantly associated with IPV types and terminated pregnancy.

**Conclusion:**

Findings indicate the role of community influence in the association between IPV-exposure and terminated pregnancy, and stress the need for screening women seeking abortions for a history of abuse.

## Background

In Nigeria, terminated pregnancy (also known as abortion) is illegal except under medical conditions to save a woman’s life [1]. Despite strict abortion laws in Nigeria, which carry a prison sentence of 7 and 14 years for the abortion seeker and provider, respectively [2], an estimated 610,000 terminated pregnancies were performed in 1996 (equivalent to 25 per 1,000 women of childbearing age); 142,000 of these resulted in complications severe enough to require hospitalization. In 2006, the number of terminated pregnancies was estimated to have risen to about 760,000 [3]. A large number of terminated pregnancies are performed under unsafe non-medical circumstances, thereby constituting a major source of maternal morbidity and mortality [3,4], estimated at about 1,100 deaths per 100,000 live births - one the world’s highest [4]. It is conservatively estimated that over 3,000 women die annually in Nigeria as a result of unsafe terminated pregnancies [5]. Studies have consistently shown that the high rates of terminated pregnancies reflect high levels of unintended pregnancies [1,6]; out of the estimated 6.8 million pregnancies occurring yearly in Nigeria, one in five is unplanned, with half of these ending in induced abortion [1].

Contraception has been widely acknowledged to be an effective means of combating unwanted pregnancy and unsafe pregnancy terminations. Contraceptive use in Nigeria is low despite the large number of women who have experienced unintended pregnancy and the increasing desire for Nigerian women and families to have smaller families – these imply high levels of unmet family planning needs in the country [1,7,8]. However, a myriad of reasons have been suggested as being responsible for the poor use of contraceptives and unmet contraceptive needs among married and unmarried women in Nigeria. These range from the high premium placed on childbearing and religious teachings [9,10], the use of a combination of modern and traditional methods of contraception [11], to the poor distribution and availability of contraceptives, and women’s fear of contraceptive side-effects [12-14].

### Introduction

The use of “any acts of physical, sexual or emotional abuse by a current or former partner whether cohabitating or not” is commonly referred to as intimate partner violence (IPV) [15]. IPV is a widespread public health problem with serious consequences for women’s physical, mental, sexual and reproductive health [15-17]. Reproductive health outcomes associated with IPV include unwanted and terminated pregnancies, fetal loss, non-use of family planning methods, and high fertility [16-20]. Recent studies from other contexts suggest a higher prevalence of IPV among women seeking elective abortions compared with women who take their pregnancies to term [21-23], and that their decision to terminate a pregnancy was influenced by their exposure to abuse [23]. Other studies attribute the lower tendency of IPV-exposed women to take pregnancy-preventive measures to fear of further abuse [24], and a higher tendency to seek termination in the occurrence of pregnancy [25]. Although the dimension of pregnancy-related violence mentioned above are integral to a more complete understanding of IPV and pregnancy outcomes, the extant literature is lacking a comprehensive contextual aspect of the community-level risk markers that may contribute to vulnerability, patterns and pregnancy outcomes associated with IPV. Existing studies that investigated associations between IPV and terminated pregnancy focused only on individual-level predictors, without investigating the role of community-level factors [19,25].

Interest in the role of community influences on the association between IPV and terminated pregnancy stems from the increasing awareness of IPV as a product of social context [26,27]. Women living in communities with prevailing traditional cultural values and gender norm are at increased risk of abuse by an intimate partner, which when abuse occurs in pregnancy, may result in terminated pregnancy. Thus, community-level characteristics are vital to women’s reproductive health – a less explored mechanism. Certain contextual aspects of various cultures strongly discourage modern contraceptive usage, such as levels of education and community attitudes toward gender roles and wife beating, may influence the risk of IPV and reproductive health outcomes [28,29]. Moreover, in some rural Nigerian communities, there is the belief that the use modern contraceptives reduces the number of children a women is destined to have, which may be punishable with infertility on re-incarnation [8]. We provide evidence of the role of community-level factors associated with terminated pregnancy - a subject that is difficult to study in contexts where abortion is severely restricted by law.

### Theoretical perspectives

**Figure 1:**
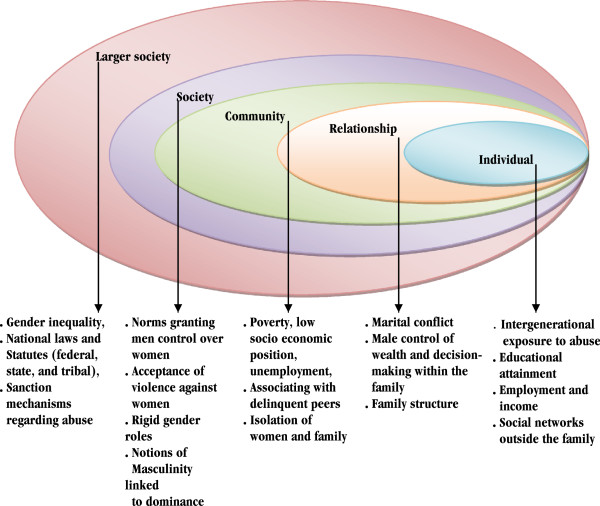
Social ecological model (adapted from Heise (1998).

The study is guided by the social ecological theory [30] (Figure 1), which facilitates understanding of the influence of factors at multiple levels (individual, dyad, family, community, and societal) on such harmful behavioral outcomes as IPV. The individual level describes individual experiences of gender norms that predispose women to abuse [31], or financial dependence [32]. The relationship level refers to interactions between couples, families and other small groups, and includes male control over family resources, decision-making autonomy, economic inequalities, and controlling behaviour by spouse or partner [33]; these factors increase the likelihood of IPV. Controlling behaviour by husband or partner significantly predispose women to abuse by acting as a contributing factor to violence. Women’s justification of traditional gender norms of wife beating by a husband/partner is strongly correlated with different forms of IPV [33,34], which is widely considered to be a consequence of accepted traditional norms and values permitting men to inflict punishment on their wife/partner, thereby promoting abuse. Relationships that embrace egalitarian decision-making and equal division of power often report low levels of conflict, control, and abuse [35]. Thus, women with greater decision-making autonomy may be perceived as defying societal gender roles and challenging their partner’s masculinity as provider or breadwinner. As a result, they become vulnerable to their partner’s control tactics to curtail such defiance, including abuse [36]. Measures of relationship inequality (e.g. spouses’ relative earning, spouses’ relative education, and spouses’ relative age) may influence the risk of abuse in certain societal contexts; the greater the equality (or less inequality) between partners, the higher the women’s risk of IPV-exposure, as this threatens certain men’s perception of power within relationships [37,38]. For instance, women who earn more than their husband/partner, or are more educated than their husband/partner may be more vulnerable to control and abusive acts [35]. The community level highlights the accepted traditions and values of members of communities aggregated at the community level [36]. Finally, societal level focuses on national laws and statutes (federal, state, and tribal) that either tolerate of discourage abusive behaviour against women [27].

An understanding of the underlying contextual factors associated with IPV and terminated pregnancy is needed in order to develop a more effective response to this social problem. Several risk markers have been investigated as they relate to a woman's likelihood of terminating a pregnancy in association with IPV. Thus, understanding the contextual factors that increase the vulnerability of certain women to terminate a pregnancy is as important as incorporating these risk markers into theoretical frameworks in order to provide a more comprehensive understanding of this association.

This study hypothesizes firstly, that IPV-exposed women are more likely to terminate a pregnancy than women not exposed to IPV. Secondly, that women exposed to forms of control or coercive behaviours by their partner have a higher likelihood of terminating a pregnancy in association with IPV; and thirdly, that women resident in empowered communities (i.e. communities with lower levels of justification of wife beating, and higher level of contraceptive use) are less likely to experience IPV or report terminating a pregnancy. Thus, this paper addresses gaps in the existing literature concerning the community-level factors that make women vulnerable to pregnancy termination following IPV. The correlates of unwanted- and terminated pregnancy per se are however outside the scope of this study. To our knowledge, no study within the Nigerian or sub-Saharan African (SSA) context has examined the role of community-level factors on the interplay between IPV and terminated pregnancy. Therefore, the aim of this study was to examine the role of community-level norms and characteristics in the association between intimate partner violence (IPV) and terminated pregnancy in Nigeria, focusing also on relationship control at the individual- and relationship levels.

## Methods

### Data and participants

This study is based on cross-sectional data from the 2008 Nigeria Demographic and Health Survey (DHS), for which details of the study design are discussed elsewhere [39]. Briefly though, data were collected by a stratified two-stage cluster sample design, with the list of enumeration areas (EAs) developed from the 2006 Population Census as the sampling frame. 888 primary sampling units (PSUs) were selected in the first sampling stage, from which 7,864 households were systematically selected in the second sampling stage using a probability sample technique.

### Data collection

Trained interviewers speaking the same language as respondents obtained verbal individual consent; respondents were informed of the objectives of the study, their right not to respond to any question they were not comfortable answering, and that they could terminate the interview at any time. Due to the sensitive nature of the questions, interviews for the domestic violence module were undertaken only when privacy was achieved; this was maintained throughout the process as supported by the World Health Organization’s ethical and safety recommendations for research on domestic violence assuring respondent’s privacy and anonymity [40].

### Study sample

**Figure 2:**
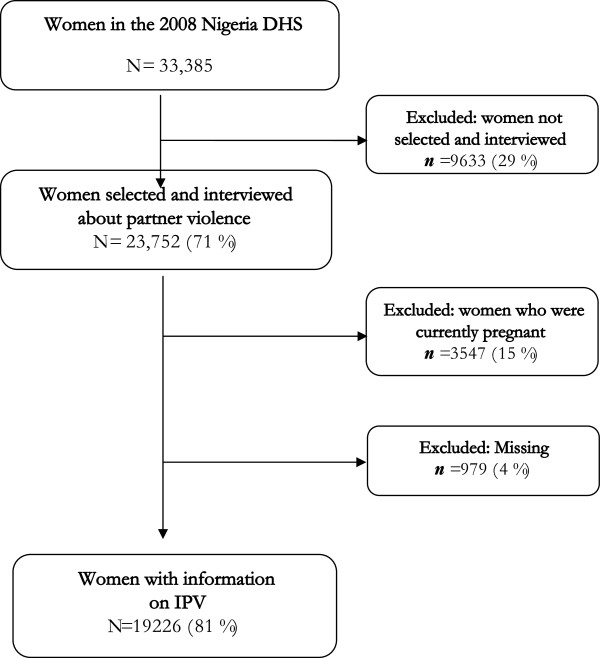
Flow chart for selection of study subjects.

Of the 33,385 women aged 15 - 49 years who participated in the study, 23,752 (71%) were randomly selected and interviewed about partner violence, and the 9,633 (29%) that were not selected and interviewed were excluded. From the selected women, 3,547 (15%) who were pregnant at the time of the interviews were also excluded. A further 979 (4%) of women were excluded for missing responses. Using the DHS domestic violence questionnaire based on a modified and previously validated version of the Conflict Tactics Scale (CTS) [41], data on IPV were therefore obtained from 19,226 women who were currently or formerly married or cohabiting with a male partner by face-to-face interviews from households selected for this purpose (Figure 2).

### Methods

*Outcome*: Terminated pregnancy - the health outcome of interest, was assessed by asking respondents the question “*have you ever had a terminated pregnancy*?” Responses were used as a dichotomous “yes” and “no” variable.

*Exposures*: (1) Intimate partner violence (IPV), used as “yes” and “no” variables created from responses to questions respondents were asked concerning their ever having experienced one or several of the following acts of abuse by a current or former husband or intimate partner: *i*) pushing, shaking or throwing something at her; *ii*) slapping her or twisting her arm; *iii*) punching or hitting her with something harmful; *iv*) kicking or dragging her; *v*) strangling or burning her; *vi*) threatening her with a weapon (e.g. gun or knife); *vii*) attacking her with a weapon; *viii*) humiliating her in public; *ix*) threatening her or someone close to her; *x*) forced sexual intercourse; and *xi*) other sexual acts when undesired. Physical violence was assessed by items (*i* – *vii*), Cronbach’s alpha (α = .90); emotional violence by items (*viii*) and (*ix*), α = .80; sexual violence by items (*x*) and (*xi*), α = .83, and any physical, sexual or emotional violence (“any IPV”) was assessed by the 11 items (*i – xi*), α = .81.

(2) Relationship control - three variables were used to assess relationship control within the Nigerian context: *i) Controlling behavior*: responses to six questions about whether present or former husband/partner had control issues: jealous if she talks with other men, accuses her of unfaithfulness, does not permit her to meet her friends, tries to limit her contact with family, insists on knowing where she is, and doesn’t trust her with money were used to create a composite dichotomous variable. Responses of “yes” to one or several of the controlling attitude questions were categorized as “yes”, whilst responses of “no” to all these questions were categorized as “no”, (α = .90); *ii) justifying wife beating*, a composite dichotomous variable created from responses to five questions enquiring whether respondents would justify partner abuse of a woman for the following reasons: if she goes out without telling him; if she neglects the children; if she argues with him; if she refuses to have sex with him; and if she burns the food. Responses of "yes" to one or several of these attitude questions were categorized as “yes”, and responses of "no" to all the attitude questions were categorized as “no”, (α = .88); and *iii*) *decision-making autonomy*, a categorical "yes" or "no" variable created from responses to five questions on whether respondents were involved in the “final say” in certain circumstances: large household purchases; daily household purchases; visits to family or friends; own health; and deciding what to do with husband’s money. Women whose response to one or several of these questions was either "respondent alone" or "respondent and husband/partner" to one or several of these questions were categorized as “yes”, whilst women whose responses to all these questions was "other person in the household" "no" were categorized as “no”, (α = .89).

(3) Relationship inequalities – assessed using three variables: *i)* spouses’ relative earnings (earns less than spouse, earns same as spouse, earns more than spouse, and spouse does not contribute); *ii)* spouses’ relative education (less educated than spouse, same education as spouse, and more educated than spouse); and *iii)* spouses’ relative age, younger than spouse, same age as spouse, and older than spouse). (4) Socio-demographic characteristics – these included: *i) women’s age* (≤ 24, 25 – 34, and ≥ 35 years); *ii*) *women’s education* (no education, primary education, and secondary or higher education); *iii*) *women’s employment* (employed, and unemployed); *iv*) *marital status* (married, and single); *vii*) *place of residence* (urban, and rural); *viii*) *household wealth index*, categorized as (poor, middle, and rich), and constructed from responses to questions about household possession of such durable items as; radio, refrigerator, television, and motorcar, quality of dwelling such as floor type or roof type, using principal component analysis (PCA); and *ix*) *use of modern contraceptive method* (yes, and no). (5) Community-level factors reflecting the social contexts in Nigeria (i.e. norms/beliefs, illiteracy) were examined using four indicators: *i) community mean education*, defined as the mean number of years of female education per woman in the community; *ii) community attitudes justifying wife beating*, defined as the percentage of women with tolerant attitudes to wife beating in the community; *iii) community median age of first marriage*, defined as mean age at first marriage for women in the community; and *iv) community contraceptive use*, defined as the percentage of women that used modern contraceptive methods within the community, categorized into low, middle, and high. Communities were identified within DHS data as PSUs, which are small and fairly homogenous administratively-defined areas representing sampling blocks of about 20 to 30 households.

#### Analysis

Cross-tabulations and Pearson’s chi-square (*χ*^2^) analyses were used to assess the differences in the association between the outcome and exposure variables, with significance level set at *p* < 0.05.

Bivariate associations between terminated pregnancy and exposure variables were determined, and only variables with significance levels α = 0.10 (Table not shown) were entered into the multilevel analysis. Multilevel logistic regression models were carried out using generalized linear and latent mixed models (gllamm) [42]; this enabled the simultaneous assessment of the association between individual and community-level factors, IPV, and terminated pregnancy. A two-level analysis with respondents (level 1) nested within PSUs (level 2) was performed using Stata 11.0 [43]. The crude analyses contained only the IPV types, which were adjusted for individual and community-level variables. Results of fixed effects (measures of association) were expressed as odds ratios (ORs) with 95% confidence intervals (95% CIs). Random effects (measures of variation, which indicate the relatedness of clustered data) were expressed as neighbourhood-level variance (σ^2^) with standard errors (SE). Intraclass correlation coefficient (ICC) at the neighbourhood level was computed in order to estimate the extent to which the propensity for terminated pregnancy for individuals within the same community was similar to that for individuals in other communities. The ICC indicates the proportion of total variance that exists between neighborhoods i.e. level 2 [44].

#### Ethical considerations

Permission to use this data in this study was obtained from ORC Macro Inc. (initial approval was obtained from the National Ethics Committee in the Federal Ministry of Health of Nigeria and the Ethics Committee of the Opinion Research Corporation Macro international, Inc. (ORC Macro Inc., Calverton, MD; USA).

## Results

### Prevalence of induced abortion

Characteristics of the respondents by occurrence of terminated pregnancy are presented in Table 1. Prevalence of terminated pregnancy was 6% (n = 150) in relation to sexual IPV, 19% (n = 483) in relation to emotional IPV, 21% (n = 530) with physical IPV, and 30% (n = 780) with any IPV. A higher proportion of the women who terminated a pregnancy earned less than their spouse (34%, n = 4155; *p* < 0.001), were more educated than their spouse (32%, n = 3394; *p* < 0.001), younger than their spouse (58%, n = 4702; *p* < 0.031), 35 years or older (47%, n = 1701; *p* < 0.0001), had secondary or higher education (36%, n = 1297; *p* < 0.000), employed (70%, n = 2623; *p* < 0.000), married (89%, n = 3218; *p* < 0.0001), did not use modern contraceptives (90%, n = 3251; *p* < 0.042), and resident in rural areas (68%, n = 2465; *p* < 0.424).

**Table 1:** Characteristics of the study population by terminated pregnancy

	**Terminated pregnancy**			
	**Yes, n (%)**	**No, n (%)**	***p*-value**	**Total, N (%)**
**Physical IPV**^**§**^			***	
Yes	530 (21)	2,366 (14)		2,896 (15)
No	2,039 (79)	14,291 (86)		16,330 (85)
**Sexual IPV**^**§**^	*n* = 2,553		***	
Yes	150 (6)	533 (3)		683 (3)
No	2403 (94)	16,140 (97)		18,543 (97)
**Emotional IPV**^**§**^	*n* = 2549		***	
Yes	483 (19)	2,227 (13)		2,710 (14)
No	2,066 (81)	14,450 (87)		16,516 (86)
**Physical, sexual or emotional**^**§**^			***	
Yes	780 (30)	3,456 (21)		4,236 (22)
No	1,812 (70)	13,178 (79)		14,990 (78)
*Spouses’ relative earnings*			***	
Woman earns less than spouse	4,155 (34)	11,849 (56)		16,004 (48)
Woman earns same as spouse	2,628 (22)	3,162 (15)		5,790 (17)
Woman earns more than spouse	2,676 (22)	2,967 (14)		5,643 (17)
Partner does not contribute	2,647 (22)	3,234 (15)		5,881 (18)
*Spouses’ relative education*			***	
Woman has less than spouse	2,864 (27)	2,975 (13)		18,566 (56)
Woman has same as spouse	4,382 (41)	14,184 (63)		5,839 (17)
Woman has more than spouse	3,394 (32)	5,519 (24)		8,913 (27)
*Spouses’ relative age*			*p* < .031 **	
Woman younger than spouse	4,702 (58)	21,474 (85)		26,176 (78)
Woman same age as spouse	1,690 (21)	1,874 (7)		3,564 (11)
Woman older than spouse	1,688 (21)	1,890 (8)		3,578 (11)
*Controlling behavior*			***	
Yes	1,645 (65)	10,378 (63)		7,013 (37)
No	868 (45)	6,145 (37)		12,023 (63)
*Justifying wife beating*			***	
Yes	1,889 (52)	14,368 (49)		16,257 (49)
No	1,720 (48)	15,136 (51)		16,856 (51)
*Decision-making autonomy*			***	
Yes	2,018 (64)	12,640 (63)		14,658 (63)
No	1,122 (36)	7,543 (37)		8,665 (37)
*Women’s age*			***	
≤ 24	576 (16)	12,082 (41)		12,658 (38)
25 - 34	1,348 (37)	9,493 (32)		10,841 (32)
≥ 35	1,701 (47)	8,118 (27)		9,819 (30)
*Women’s education*			***	
No education	1,468 (40)	11,739 (40)		13,207 (40)
Primary school	860 (24)	5,721 (19)		6,581 (20)
Secondary school or higher	1,297 (36)	12,233 (41)		13,530 (40)
*Employment status*			***	
Unemployed	1,108 (30)	12,661 (43)		13,769 (41)
Employed	2,623 (70)	16,926 (57)		19,549 (59)
*Marital status*			***	
Married	3,218 (89)	20,691 (70)		23,909 (72)
Single	407 (11)	9,001 (30)		9,408 (28)
*Modern contraceptive use*			*p* < .042	
Yes	374 (10)	2,753 (9)		3,127 (9)
No	3,251 (90)	26,940 (91)		30,191 (91)
*Place of residence*			*p* < .424	
Urban	1,160 (32)	9,308 (31)		10,468 (31)
Rural	2,465 (68)	20,385 (69)		22,850 (69)

^**§**^ Data from women selected for domestic violence assessment.

ADD mean age at first marriage, mean educationallevel.

### Association between terminated pregnancy and IPV types

Table 2 shows the crude and adjusted ORs of the association between terminated pregnancy, the different types of IPV, and explanatory variables. The crude ORs showed significant associations between terminated pregnancy and all the forms of IPV among IPV-exposed women compared to non-exposed women. After adjusting for individual- and community-level factors, with the exception of emotional IPV, the likelihood of terminated pregnancy was higher among women exposed to physical IPV (OR = 1.52, 95% CI: 1.21 - 1.91), sexual IPV (OR = 1.62, 95% CI: 1.07 - 2.44), and “any” IPV (OR = 1.44, 95% CI: 1.19 - 1.76) compared to non-exposed women.

**Table 2:** Multilevel logistic regression models for the association between terminated pregnancy and single entity IPV against women

**Characteristics**	**Terminated pregnancy**							
	**Physical IPV**		**Sexual IPV**		**Emotional IPV**		**Any IPV**	
	**Crude**	**Adjusted**	**Crude**	**Adjusted**	**Crude**	**Adjusted**	**Crude**	**Adjusted**
	**OR (95% CI)**	**OR (95% CI)**	**OR (95% CI)**	**OR (95 % CI)**	**OR (95 % CI)**	**OR (95% CI)**	**OR (95% CI)**	**OR (95% CI)**
*Physical IPV*								
Yes (No = reference)	1.46 (1.30 – 1.65)	1.52 (1.21 – 1.91)	-	-	-	-	-	-
*Sexual IPV*								
Yes (No = reference)	-	-	1.60 (1.30 – 1.97)	1.62 (1.07 – 2.44)	-	-	-	-
*Emotional IPV*								
Yes (No = reference)	-	-	-	-	1.39 (1.23 – 1.56)	1.21 (0.97 – 1.57)	-	-
*Physical, emotional or sexual IPV*								
Yes (reference: No)	-	-	-	-	-		1.50 (1.35 – 1.66)	1.44 (1.19 – 1.76)
*Controlling behavior*								
Yes		3.25 (2.62 – 4.02)		4.02 (2.50 – 6.47)		2.74 (2.24 – 3.34)		3.02 (2.54 – 3.60)
No		1		1		1		1
*Justifying wife beating*								
Yes		1.59 (1.31 – 1.94)		2.13 (1.43 – 3.17)		1.21 (1.00 – 1.46)		1.37 (1.16 – 1.62)
No		1		1		1		1
*Decision-making autonomy*								
Yes		1.31 (1.06 – 1.63)		1.63 (1.06 – 2.51)		1.00 (0.82 – 1.23)		1.14 (0.95 – 1.37)
No		1		1		1		1
*Spouses’ relative earnings*								
Woman earns less		1.08 (0.74 – 2.59)		2.53 (0.87 – 7.35)		1.26 (0.84 – 1.90)		1.04 (0.74 – 1.46)
Woman earns same		1		1		1		1
Woman earns more		1.33 (0.80 – 2.22)		5.49 (1.67 - 18.01)		2.23 (1.32 – 3.75)		1.56 (1.00 – 2.44)
Partner does not contribute		0.72 (0.41 – 1.26)		2.51 (0.69 – 9.19)		3.42 (2.09 – 5.60)		2.11 (1.38 – 3.24)
*Spouses’ relative education*								
Woman has less		1.02 (0.35 – 2.92)		0.61 (0.07– 5.18)		1.62 (0.72 – 3.66)		1.51 (0.70 – 3.23)
Woman has same		1		1		1		1
Woman has more		1.15 (0.91 – 1.44)		1.60 (1.03 – 2.48)		1.25 (0.49 – 1.59)		1.17 (0.96 – 1.43)
*Spouses’ relative age*								
Woman younger		1.58 (0.69 – 3.60)		1.36 (0.29 – 6.35)		1.47 (0.67 – 3.25)		1.77 (0.87 – 3.58)
Woman same age		1		1		1		1
Woman older		0.75 (0.21 – 2.65)		0.51 (0.04 – 6.77)		1.15 (0.37 – 3.58)		1.16 (0.42 – 3.24)
*Women’s age*								
≤ 24		1.12 (0.86 – 1.45)		1.71 (1.05 – 2.78)		0.89 (0.70 – 1.13)		0.98 (0.79 – 1.21)
25 - 34		1.34 (1.10 – 1.62)		1.39 (0.94 – 2.06)		0.92 (0.76 – 1.11)		1.03 (0.87 – 1.21)
≥ 35		1		1		1		1
*Women’s education*								
No education		0.75 (0.55 – 1.02)		0.97 (0.53 – 1.76)		0.96 (0.71 – 1.29)		0.82 (0.63 – 1.07)
Primary school		0.64 (0.49 – 0.83)		0.67 (0.41 – 1.11)		0.60 (0.46 – 0.78)		0.65 (0.52 – 0.82)
Secondary school or higher		1		1		1		1
*Employment status*								
Unemployed		0.79 (0.48 – 1.29)		0.70 (0.26 – 1.90)		0.47 (0.30 – 0.73)		0.53 (0.36 – 0.78)
Employed		1		1		1		1
*Marital status*								
Married		0.95 (0.79 – 1.16)		0.92 (0.76 – 1.21)		0.96 (0.79 – 1.17)		0.96 (0.79 – 1.17)
Single		1		1		1		1
*Modern contraceptive use*								
Yes		1		1		1		1
No		1.55 (1.21 – 1.73)		2.63 (1.39 – 3.88)		1.04 (0.84 – 1.85)		1.54 (0.74 – 1.70)
*Place of residence*								
Urban		0.55 (0.41 – 0.73)		0.53 (0.31 – 0.88)		0.64 (0.48 – 0.85)		0.54 (0.42 – 0.70)
Rural		1		1		1		1
*Community mean education*		1.12 (1.06 – 1.20)		1.09 (0.97 – 1.22)		1.14 (1.07 – 1.21)		1.16 (1.10 – 1.23)
*Community justify wife beating*								
Low		0.70 (0.52 – 0.94)		0.90 (0.51 – 1.58)		0.78 (0.58 – 1.05)		0.75 (0.57 – 0.97)
Median		1		1		1		1
High		0.98 (0.73 – 1.30)		1.46 (0.86 – 2.48)		0.43 (0.69 – 1.24)		0.84 (0.64 – 1.09)
*Community mean age of marriage*		1.02 (0.95 – 1.10)		1.09 (0.96 – 1.25)		0.90 (0.83 – 0.97)		0.91 (0.85 – 0.97)
*Community contraceptive use*								
Low		1.64 (1.12 – 1.92)		1.39 (1.14 – 2.06)		1.02 (0.86 – 1.18)		1.03 (0.87 – 1.23)
Median		1		1		1		1
High		0.75 (0.69 – 0.95)		0.71 (0.46 – 0.93)		0.80 (0.57 – 1.28)		0.65 (0.62 – 0.82)
**Random effects**								
Neighbourhood variance (SE)	1.038 (0.083)	0.865 (0.140)	1.362 (0.172)	1.550 (0.389)	0.708 (0.062)	1.032 (0.149)	0.799 (0.060)	0.899 (0.119)
Intra-class correlation (ICC)	0.240	0.208	0.293	0.320	0.177	0.239	0.195	0.215

### Association between IPV types and explanatory factors

All the IPV types were significantly associated with factors reflecting relationship control, such as having a husband/partner who exhibited controlling behaviour compared to husband/partner without controlling behaviour, justifying wife beating (with the exception of exposure to emotional IPV) compared to not justifying wife beating, and decision-making autonomy (with the exception of exposure to emotional IPV and “any” IPV) compared to not having decision-making autonomy. Among the variables assessing relationship inequalities, women earning more than their spouse (OR = 5.49, 95% CI: 1.67 - 18.01), and women who were more educated than their spouse (OR = 1.60, 95% CI: 1.03 - 2.48) had a significantly higher likelihood of exposure to sexual IPV in comparison with those who earned the same as their spouse and had the same level of education as their spouse, respectively. Emotional IPV was significantly associated with women earning more than their spouse (OR = 2.23, 95% CI: 1.32 - 3.75), and women whose partners did not contribute to household earnings (OR = 3.42, 95% CI: 2.09 - 5.60) compared to those who earned the same as their spouse. “Any” IPV was significantly associated with women having partners who did not contribute to household earnings (OR = 2.11, 95% CI: 1.38 - 3.24) in comparison to those who earned the same as their spouse. Relationship age differences were not statistically significantly associated with any of the IPV types.

Women who were ≤ 24 years (OR = 1.71, 95% CI: 1.05 - 2.78) were significantly more likely to experience sexual IPV compared to women 35 years or older, whilst women 25 – 34 years (OR = 1.34, 95% CI: 1.10 - 1.62) were significantly more likely to experience physical IPV compared to women 35 years or older. Women with primary education had a significantly lower likelihood of experiencing physical IPV (OR = 0.64, 95% CI: 0.49 - 0.83), emotional IPV (OR = 0.60, 95% CI: 0.46 - 0.78), and “any IPV” (OR = 0.65, 95% CI: 0.52 - 0.82) compared to those who had secondary or higher education. Unemployed women had a significantly lower likelihood of experiencing emotional IPV (OR = 0.47, 95% CI: 0.30 - 0.73), and “any IPV” (OR = 0.53, 95% CI: (0.36 - 0.78) compared to employed women. Women who did not use modern contraceptives had a significantly higher likelihood of experiencing all the IPV types compared to those who used modern contraceptives. Women resident in urban areas were significantly less likely to experience all the IPV types compared to women resident in rural areas. The association between marital status and IPV types was not statistically significant.

Among the community-level factors, having a level of education above the mean for the community was associated with a higher likelihood of experiencing physical IPV (OR = 1.12, 95% CI: 1.06 - 1.20), emotional IPV (OR = 1.14, 95% CI: 1.07 - 1.21), and “any IPV” (OR = 1.16, 95% CI: 1.10 - 1.23). Residence in a community with justification of wife beating below the median for the community was associated with a lower likelihood of women experiencing physical IPV (OR = 0.70, 95% CI: 0.52 - 0.94), and any IPV (OR = 0.75, 95% CI: 0.57 - 0.97) compared to residence in a community with justification of wife beating at the median for the community. Marrying at the mean age of marriage for the community was associated with a lower likelihood of women experiencing emotional IPV (OR = 0.90, 95% CI: 0.83 - 0.87), and “any IPV” (OR = 0.91, 95% CI: 0.85 - 0.97), whilst living in a community with modern contraceptive use below the median for the community was associated with a higher likelihood of women experiencing physical IPV (OR = 1.64, 95% CI: 1.12 - 1.92), and sexual IPV (OR = 1.39, 95% CI: 1.14 - 2.06) compared to residence in a community with modern contraceptive use at the median for the community. Finally, the variance between neighbourhoods was consistently more than twice the standard error for the adjusted models with regards to all the IPV types, indicating significant differences between neighbourhoods in the likelihood of terminating a pregnancy. The ICC for terminated pregnancy in the adjusted models was 0.208 for physical IPV, 0.320 for sexual IPV, 0.239 for emotional IPV, and 0.215 for any IPV, indicating that 21% (physical IPV), 32% (sexual IPV), 24% (emotional IPV), and 21% (any IPV) of the total variance in terminated pregnancy in relation to these forms of IPV could be explained at the neighbourhood or community level.

## Discussion

### Summary of results

This study examined the role of community influences on women's experience of intimate partner violence and terminated pregnancy in Nigeria. Findings include: first, that among women who had terminated a pregnancy, there was a lifetime prevalence of physical IPV (21%), sexual IPV (6%), emotional IPV (19%), and “any IPV” (30%); second, that different forms of IPV were significantly associated with terminated pregnancy; third, that community-level factors influenced the association between women’s exposure to IPV and terminated pregnancy; fourth, that indicators of relationship control were significantly associated with various forms of IPV and terminated pregnancy; and fifth, that characteristics reflecting relationship inequalities explained some of the association between forms of IPV and terminated pregnancy.

### Comparison with other studies

The lifetime prevalence of the different IPV types associated with terminated pregnancy among women found in this study is comparable to findings in studies in Bangladesh and Canada [45,46], but comparatively lower than those in a study in Kenya [19]. These differences may be methodological and linked to varied understanding of the term “terminated pregnancy”, and under-reporting of both terminated pregnancy and IPV within the different country contexts. Women who had terminated a pregnancy were more likely to have experienced physical IPV, sexual IPV, and “any IPV” compared to women who had no prior experience of these IPV types, which is consistent with findings from studies in other national contexts [19,24,26,47-49], and validates the first hypothesis in this study. These findings may be partly attributed to the characteristics of the women that had terminated a pregnancy, given that the women were more often rural residents, did not use contraceptives, were younger than their spouse, earned less than their spouse, and had a spouse who was more likely to have controlling behaviour (corresponding to several explanatory factors listed in Table 1). As such, some of these women may have been economically dependent on their spouse, and lacked greater control over their fertility by not being empowered enough to refuse sex or negotiate the use of contraceptives for fear of abuse [50,51]. The women may also have been more prone to experience IPV due to the general acceptance of male dominance within the societal contexts within which they live in, which enforce more traditional gender roles. It is also plausible that women who are exposed to IPV might have less control over the choice of contraceptive method, and not taking contraceptives for fear of further abuse [25], and are more likely to seek termination in the event of pregnancy [24,26,46]. Our findings however contrast with those in a study based on men’s self-reported perpetration of abuse in Bangladesh [45], which showed a non-significant association between “any IPV” and terminated pregnancy. Emotional IPV was however not significantly associated with terminated pregnancy after adjusting for confounders in this study, which is in line with a study from Cameroon [52]. Further in depth research is needed into the association between physical and non-physical or non-sexual IPV and pregnancy outcomes.

Of the measures of relationship control, controlling behavior by husband/partner was consistently the strongest factor associated with all the IPV types and terminated pregnancy, which corroborates findings from a recent study [53], and indicates that male partner controlling behaviour plays a significant role in the occurrence of IPV. This may stem from the need for abusive male partners to enforce power and control within the relationship and during pregnancy. This finding is consistent with those from the Nigerian [33], and other contexts documenting women being coerced by abusive partners to have abortions [54,55]. We presume that abusive and controlling male partners may influence their female partner’s non-use of contraceptives, and in the event of pregnancy, may exhibit behaviours (including violent acts) to control pregnancy outcomes in an attempt to induce abortion or coerce women to terminate the pregnancy [56]. This stresses the need for further population-based research investigating forms of reproductive coercion and associations with IPV. Likewise, longitudinal studies are needed to examine the interrelation between reproductive coercion, IPV and unintended/terminated pregnancy, possibly specifying the appropriate chronological order and patterns, so as to better inform the design of interventions to reduce both the risk for unintended/terminated pregnancy and IPV victimization.

In related findings, we found a positive association between women justifying wife beating and IPV (physical; sexual; and “any”), consistent with another other studies [7,33]. This symbolizes women’s acceptance of violence towards them within societies that tolerate conventional gender role attitudes that permit men to discipline their wife/partner for various transgressions. These attitude of accepting wife beating increases the likelihood of IPV, and within the context of abusive relationships, may be accompanied by pregnancy coercion and termination when they occur in pregnancy [56]. The finding that having decision-making autonomy was associated with IPV (physical; and sexual) may represent the indirect causal mechanisms through which IPV affects women’s reproductive health outcomes [25,57]. These collectively indicate the skewed power imbalances in intimate relationships within patriarchal societies where women’s subordination and submission to men is expected, accepted, and in many cases, attractive to some men [51], thus supporting the second hypothesis in this study, and validates those reported by others [54-57]. Our finding therefore calls for policies that would enforce women’s reproductive autonomy (i.e. women’s ability to make independent decisions about their reproduction).

The significance of intra-relationship economic power in the dynamics of abuse and terminated pregnancy is evident in the finding that women who earned more than their spouse, and women who were more educated than their spouse were more likely to experience sexual IPV; likewise, women who earned more than their spouse, and women whose spouse did not contribute to household earnings were more likely to experience emotional IPV (though the association between emotional IPV and terminated pregnancy was statistically non-significant), and women whose spouse did not contribute to household earnings were more likely to experience “any IPV”. Some of these findings have been previously reported [27,58], although only in relation to the association between relationship characteristics and IPV. Differences in relationship characteristics have not been fully explored in relation to such outcomes as terminated pregnancy, and to our knowledge, this is the first study to examine this relationship. Plausible explanations are that women who are earn more or are more educated than their male partner in this context may be regarded as challenging existing gender norms, which increases the likelihood of exposure to abuse and the possibility, and also shown in other studies [59,60]. This calls for behavioural change interventions promoting joint decision-making within intimate relationships as an attractive strategy for increasing women’s views within the marriage whilst encouraging men to settle household disputes through negotiation, and not violence.

Among the socio-demographic explanatory factors, the finding that women 25–34 years were more likely to report physical IPV, and women 24 years or younger were more likely to report sexual IPV in comparison to women 35 years or older is in agreement with findings from other studies [26,61], which indicate that younger age at pregnancy is associated with higher risk of partner violence. Younger women tend to be less educated, which may translate to limited economic opportunities, increased vulnerability, and economic dependence on the male partner, and submission to male dominance and abuse [62]. Alternative explanations may be under-reporting of abuse among women 35 years or older who tend to be more educated, have higher socioeconomic status within and beyond the household, and greater reproductive autonomy, and possibly not desiring more children, wanting instead to pursue a career. These findings substantiates those from a study documenting high rates of abuse in intimate relationships among women (especially younger women) presenting for sexual and reproductive health services [63], and underscores the potential opportunity provided by fertility or family planning clinics in initiating screening and providing intervention measures aimed at adverse reproductive outcomes (unwanted/terminated pregnancies, morbidity/mortality) and partner abuse.

Interestingly, we also found a protective effect of low socio-economic position i.e. women with primary education being less likely to report physical IPV, emotional IPV, and “any IPV”, and the likelihood of emotional IPV, and “any IPV” being lower among unemployed women. Though counter intuitive, this finding contrasts those in other studies [28,64], in which women with lower levels of education (primary or none) were shown to have an increased likelihood of IPV compared to women with higher education. Findings in our study could have resulted from under-reporting of IPV and terminated pregnancy by less educated women, given that several studies have indicated this to be the case among women in Nigeria [33,65]. As such, less educated women may be even more likely to under-report IPV due to cultural hindrances and beliefs, and a greater adherence to traditional gender norms. This emphasizes the complexity of the association, and the paradoxical effect of women’s education on IPV. Accordingly, the greater personal and economic independence associated with women with higher education may translate into perceptions of powerlessness among some men, who may become abusive, as has been reported in other studies [53,66,67]. Further research is warranted on how educational attainment in individual and community-levels is associated with IPV.

The non-use of contraceptives among women who reported physical IPV and sexual IPV is in line with findings in other studies [24,68,69], but contrasting with others [45,52,70,71]. Women in violent relationships may have reduced control over the choice and use of reproductive services, including family planning or other fertility control measures for fear of further violence from abusive partners. Alternative explanations may be related with the current economic hardship in Nigeria which may reduce the desire of some men for more children; a woman’s non-use of contraceptive may further stress abusive men to control pregnancy outcomes either directly through IPV or indirectly by coercing abused women to terminate the pregnancy [56]. Rural women were more likely to have experienced all the IPV types, which is consistent with previous studies [65]. Although rural areas are not homogenous, apart from running the risk of under-reporting, they tend to be communities with more traditional gender views [57,72]. However, a study in Nigeria indicated the higher likelihood of pregnancy terminations in urban areas [7]; differences in findings may be methodological, as our study also accounted for variations in community-level characteristics. Geographic and social isolation among rural women may also limit opportunities for IPV-exposed rural women to seek much needed social and reproductive health services [18,32,73].

Support in part for our third hypothesis is evident in the finding that women’s mean educational level for the community was associated with a higher likelihood of physical IPV, emotional IPV, “any IPV”, and terminated pregnancy. A reasonable explanation may be that women’s increased education might improve their socio-economic status and economic resources to levels equaling or surpassing that of their male partners. Abusive men within communities tolerant of IPV may perceive such women as opposing traditional values that threaten men’s role as “bread winners” of the family, resulting in higher likelihood of abuse, and terminated pregnancy. Similar findings have been previously reported [50,53], and stress the need to change traditional gender norms based on social learning processes and imitation that could be achieved by repeated social interactions and exchanges defining the parameters of acceptable behavior within communities.

The association between women’s low level of justification of wife beating within the community, physical IPV, “any IPV”, and terminated pregnancy reflects community-level norms and attitudes that accept or are indifferent to IPV and gender inequality; this is consistent with prior findings [28,29,53,74]. That age at first marriage aggregated at the community level was associated with lower likelihood of physical IPV, Sexual IPV, and terminated pregnancy is not unexpected. Early marriage is frequently associated with increase fertility [75], early childbearing and adverse outcomes for the woman, her fetus or offspring [76]. Early motherhood often terminates women’s educational possibilities, and reduces their employment opportunities, making them socio-economically dependent on their male partner. Such young women are less likely to report IPV, and in the event of pregnancy, may lack sufficient reproductive autonomy to terminate an unwanted pregnancy. Since customs surrounding marriage in Nigeria, such as the age of marriage and selection of a spouse depends to a great extent on societal norms and traditions, age at first marriage could be described as a product of society. In the light of the mounting evidence against it, there is a need for culturally-sensitive interventions aimed at changing such practices.

In addition, living in a community with levels of contraceptive use below the median for the community was associated with a higher likelihood of women’s exposure to physical IPV, sexual IPV, and terminated pregnancy; conversely, residing in a community with contraceptive use at levels above the median for the community was associated with lower likelihood of women’s experience of physical IPV, sexual IPV, “any IPV”, and terminated pregnancy. The inverse association between contraceptive use and IPV is indicative of its complexity, and reiterates our findings at the individual level. However, it is plausible that fear of abuse may constrain the ability of economically disadvantaged women to effectively negotiate or use contraception [77]. Although we cannot infer that the direction of causality flows from domestic violence to contraceptive behavior due to the cross-sectional design of this study, contraceptive use has been reported in a study in Uganda to lead to abuse [47], due to the belief that women's discrete use of contraceptives was an indication of sexual promiscuity, and a justifiable reason for abuse. Another study in South Africa found that young women attending family planning clinics were often physically abused by their partners [78]. We suggest that there is a need to actively refute common erroneous local beliefs.

Finally, the significant variance between neighbourhoods in the likelihood of terminated pregnancy associated with all the forms of abuse indicates that the total variance in terminated pregnancy in association with these forms of IPV could be explained at the neighbourhood level.

### Strengths and limitations

These findings should be interpreted in light of the following limitations. First, the cross-sectional design of the study precludes causal inference, and the assessment of temporality of the relationship between IPV and terminated pregnancy. Second, assessment of IPV is based on self-reporting and therefore subject to non-systematic errors in recall, and systematic non-disclosure; both types of error carry the risk of some exposure misclassification. Third, given that abortion is legally restrictive within the context of Nigeria, the use of indirect questioning techniques would have been more appropriate in elucidating responses to the question on pregnancy termination. Fourth, the potential for underreporting is an important concern in research on IPV due to the sensitivity of the subject, social stigma and respondent’s privacy and safety concerns. Fifth, disclosure rates of health consequences may not to be representative of the magnitude of the problem due to cultural factors. Sixth, drawbacks of using measures aggregated to the community level include possibility of misclassifying individuals into inappropriate administratively defined boundaries (PSUs), which may generate information bias; it may also disregard the heterogeneity of responses within a given municipality by summarizing the responses as single averaged responses (however, intraclass correlation for each of the community-level variables tested prior to aggregation of responses showed significant clustering of responses by communities – this significant clustering provided support for aggregating the data in this manner). Seventh, other socio-cultural confounders (e.g. risky behaviours, substance abuse, alcohol abuse) not controlled for in this study may exist that might influence the association between IPV and terminated pregnancy. Despite these limitations, our study provides evidence of the need to consider community-level factors, especially those related to gender inequality, in studies on women’s reproductive health. Others include the use of a large sample size, the nationally representative country-level data, the simultaneous assessment of individual- and community-level factors, the extended scope of the traumatic physical consequences assessed, and the adherence to stringent ethical rules when collecting DHS data on domestic violence, and the contribution of findings not previously assessed.

## Conclusions

This study provides several important findings. It confirms that there is a strong significant association between women’s experience of intimate partner violence (with the exception of emotional IPV) and terminated pregnancy; it suggests the contributory role of factors reflecting relationship control, relationship inequalities, and socio-demographic characteristics in exposure to IPV and terminated pregnancy; and it provides evidence of the role of context-specific community-level factors in the occurrence of IPV and terminated pregnancy as a consequence. It is of overriding importance to emphasize here is that context matters. For those wishing to understand and/or intervene in abuse relationships, appropriate social and cultural norms must also be taken into consideration. There is a need to screen all women seeking abortions for a history of abuse, targeting family planning and intervention programmes and policies for combating interpersonal violence at communities with high risks for intimate partner violence and with traditional gender norms so as to enhance the safety of women and the promotion of fertility control and women’s reproductive health.

## Details of ethics approval

This study is based on analysis of secondary data with all participant identifiers removed. The survey procedure and instruments used have received ethical approval from the National Ethics Committees of the respective countries and the Ethics Committee of the Opinion Research Corporation Macro International, Inc. (ORC Macro Inc., Calverton, MD, USA). Permission to use the DHS data in this study was obtained from ORC Macro Inc.

## Competing interests

The authors declare that they have no competing interests.

## Authors' contributions

DA designed the study, analyzed and interpreted the data, drafted the manuscript, reviewed and edited the manuscript. SA interpreted the data, reviewed and edited the manuscript. All authors read and approved the final manuscript.

## Pre-publication history

The pre-publication history for this paper can be accessed here:

http://www.biomedcentral.com/1471-2393/12/128/prepub
